# What are the most common controversial clinical issues in fertility preservation? A content analysis of a collaborative professional online consultation group

**DOI:** 10.1186/s12958-023-01122-5

**Published:** 2023-08-24

**Authors:** Avi Tsafrir, Avi BenHaroush, Jordana Hyman, Gilad Karavani, Tal Imbar

**Affiliations:** 1 IVF unit, Department of Obstetrics and Gynecology, Shaare Zedek Medical Center, Jerusalem, Israel; 2 Faculty of Medicine, Hebrew University of Jerusalem, Jerusalem, Israel; 3 IVF unit, Rabin Medical Center, Petah Tikva, Israel; 4 Sackler Faculty of Medicine, Tel Aviv University, Tel Aviv, Israel; 5 Fertility preservation unit, Department of Obstetrics and Gynecology Hadassah Medical Center, Jerusalem, Israel

## Abstract

**Research question:**

Clinicians involved in fertility preservation (FP) are often required to make prompt and consequential decisions despite the absence of evidence-based data. We established a collaborative professional online consultation group for fertility preservation issues. We sought to determine the main controversial clinical issues in FP as raised by participants of this group.

**Design:**

Content analysis of a dedicated community of practice interacting via a messaging application (WhatsApp) and a survey of group participants.

**Results:**

Between January 2019 and July 2022, group members posed 39 clinical questions which were discussed and debated by the group. Common themes included management of oncofertility cases (33%), potential gonadotoxicity of various therapies (23%), fertility preservation in women and girls with premature ovarian insufficiency (POI) (18%), and technical aspects of ovarian tissue cryopreservation (10%). All but one query received prompt response (mean time for first response for 95% of queries 7.1 ± 9.0 min) from a mean of 5.4 ± 3.2 members.

An anonymous online survey of group members was conducted during August 2022 (*n* = 31, response rate 94%). The majority of respondents stated they gained knowledge and assistance in clinical decision making from participation in the discussion group (90% and 58% of respondents, respectively).

**Conclusions:**

Management of clinical oncofertility cases, potential gonadotoxic effect of therapeutics and fertility preservation in women and girls with POI were the most common controversial issues in our fertility preservation community of practice. Intra-professional collaborative communication via a messaging application can aid in clinical management of fertility preservation and augment clinician’s knowledge.

**Supplementary Information:**

The online version contains supplementary material available at 10.1186/s12958-023-01122-5.

## Introduction

Fertility preservation is an emerging field in reproductive medicine. While embryo cryopreservation is well established since the 1980’s, oocyte and ovarian tissue cryopreservation have only been recognized as an effective tool for fertility preservation in the last 15 years [1, 2]. Studies have shown that future reproduction is extremely important to young cancer patients and their parents, facing treatments that may potentially affect their fertility (under the term Oncofertility) [3]. Fertility preservation for nonmalignant conditions such as endometriosis, as well as planned oocyte cryopreservation (to prevent age related fertility decline) have also gained popularity in recent years. Owing to technological advancements offering more options for fertility preservation, clinical scenarios requiring challenging decisions are rising.

Optimally, clinical decisions are based on the best available evidence where the highest quality of evidence arises from prospective, randomized clinical trials and meta-analysis of such studies. The next level of evidence is from retrospective data, followed by expert opinion. However, data from both prospective and retrospective studies accumulate slowly, and clinical dilemmas requiring prompt solutions cannot always be grounded in published evidence.

Management of patients requiring fertility preservation is often complicated and urgent. Also, the lag between the fertility preservation procedure and the eventual use of the tissue, and desired outcome – live birth—makes research challenging since knowledge regarding the efficacy of procedure is limited. Certain fertility preservation decisions, in acute life-threatening conditions, have to be made promptly, before the commencement of potentially gonatodoxic chemotherapy. Often, there is only time for a single attempt of an FP procedure, within a very limited time frame. As oncology treatments are rapidly evolving, knowledge regarding fertility consequences of novel drugs and treatments is limited. For example, targeted anti-cancer therapies, recently introduced into practice, are expected to be less gonadotoxic compared with traditional chemotherapy but little is known about the potential negative effect of such agents on future fertility [4].

Medical practice is optimally based on scientific and clinical research, and rigorous evidence-based guidelines as well as the received wisdom of experienced mentors. Since the current generation of clinicians is the first to actively practice FP, there are both few guidelines and few experienced clinicians in this area, especially in smaller units. Knowledge in medicine is traditionally gained through literature, scientific meetings (live or virtual), and clinical mentoring. In recent years, social networking applications have enabled asynchronous communication by large numbers of people. Similarly “wisdom of crowds”, enjoyed via social media, has been thought to be helpful when specialized communities of practice actively consult with multiple colleagues in the same field via applications such as Whatsapp. Indeed, this technology has proven useful in medicine for both clinician-patient communication [5] and between professionals [6]. This method may be particularly useful for niche medical disciplines involving a small number of professionals who could benefit from timely consultation with each other. Here, we report a descriptive, retrospective pilot study on a collaborative intra-professional WhatsApp group for clinical consultation and communication regarding FP.

## Methods

A WhatsApp group was founded by one of the authors in 2015 and included several clinicians with a particular interest in FP in Israel. Participation in the group was voluntary and a group administrator invited Reproductive, Endocrinology and Infertility (REI) clinicians with interest in FP to subscribe. Invitation was based on personal or professional acquaintance. There was no systematic approach to all members of a certain organization or facility, but every REI professional who requested to join the group was accepted. Participants were identified by their name within the platform. Gradually, more clinicians joined the group, and it grew from 6 to 33 physicians. All participants are certified OBGYN specialists, practicing REI in hospitals in Israel.

In Israel (population 9M), there are 26 in-vitro fertilization (IVF) units, of which 22 are part of public hospitals. Oncological treatments in Israel are provided in general hospitals and covered by national health insurance. When applicable, such patients are referred for consultation regarding FP usually within the same hospital. Both ovarian tissue cryopreservation (OTC) and oocyte/embryo cryopreservation are covered by national health insurance for patients anticipated to receive gonadotoxic treatments. Since the late 1990’s OTC has been performed in several IVF units in Israel [7, 8].

We analyzed activity in this group starting from January 2019 until July 2022. All participants in this group were identified by their full name. A search of the online chats was performed by the authors. Analysis included all discussions regarding clinical issues. Notifications, sharing of publications and regulatory issues were excluded from analysis. Two of the authors classified each discussion to a relevant clinical area and analyzed the number of responses and timing of first and last response. In addition, in August 2022 we conducted an anonymous online survey among group members regarding their experience of participating in this Whatsapp group.

### Statistical analysis

For all ratio variables, means and standard deviations were calculated, and Mann & Whitney test was carried out to compare outcomes between those who raised questions and those who did not. In addition, a Pearson correlation was carried out for the correlation between seniority and 'Assistance in clinical decision-making' and 'Knowledge in the field of fertility preservation'.

Data were prepared in Microsoft Excel, and statistical analyses were conducted using the SPSS statistical software (Version 21). The criterion for significance was Alpha (α) = 0.05 (two-sided).

The survey did not involve human research, and individual patient data were not collected. Hence, the Ethics Committee in Shaare-Zedek Medical Center, Jerusalem, Israel, confirmed that Institutional Review Board authorization was not required for this study.

## Results

In August 2022, the group comprised 33 clinicians from 18 centers in Israel (69% of all IVF units). This included REI specialists from all six large hospitals and from nine of eleven medium size hospitals in Israel, as defined by the Israeli ministry of health.

### Content analysis

Between January 2019 and July 2022, 39 clinical discussions were initiated by queries posed by group members (approximately 1 per month). Common themes included management of oncofertility cases (30% of all clinical discussions), potential gonadotoxicity of targeted anti-cancer therapies (23%), fertility preservation in women and girls with premature ovarian insufficiency (POI, 16%), and specific issues in ovarian tissue cryopreservation (9%). Examples of questions are presented in Table 1 (a full list is detailed in Supplementary Table 1). All but one query (98%) received responses immediately (mean time for first response for 95% of queries 7.1 ± 9.0 min. Mean number of responses per discussion was 14 ± 12. Mean number of responders per discussion was 5.4 ± 3.2.

**Table 1:** Examples of queries raised by group members. FP-fertility preservation. POI-premature ovarian insufficiency

Oncofertility cases (30%),
• 16.5 y.o. with Hodgkin’s disease. planned to receive 2.5 gr of cyclophosphamide. Oncologist against FP. Should we offer FP?
• 28 y.o. single male. Stage IV Hodgkin’s disease with superior vena cava syndrome. Very few immotile sperm cell in ejaculate. Should we attain testicular sperm?
Potential gonadotoxic damage of various medical treatments (23%)
• Is FP indicated in a patient with carcinoma of thyroid prior to radiotherapy?
• 23 y.o. stage IV clear cell carcinoma of kidney, planned to receive Lenvatinib plus Pembrolizumab. Should we offer FP?
Women and girls with POI (16%)
• 14 y.o. family history of POI (mother & aunt), AMH 0.6, no genetic diagnosis. Should we offer FP?
• 12.5 y.o.Turner mosaicism, low ovarian reserve markers. What to do?
Ovarian tissue cryopreservation (9%)
• how long can we keep ovarian tissue in media prior to freezing?

### Representative discussions

#### Case 1

A physician posted a query regarding management of a 30 year-old female with Hodgkin’s disease diagnosed one week after giving birth. She had a large mediastinal mass and brachial vein thrombosis, and the hematological team advised prompt commencement of chemotherapy. The physician requested advice on FP in this situation.

This query received responses from 15 group members within 2 h of posting. The ensuing discussion considered treatment and timing options and specific risks for this patient. Some physicians recommended ovarian stimulation and embryo cryopreservation and expressed varying opinions concerning the probable limited response to gonadotropins in the immediate postpartum period, the choice of gonadotropins, and the recommended ovulation trigger with hCG. Several participants noted potential complications of anesthesia due to the mediastinal mass and the risk of hormonal stimulation exacerbating the brachial vein thrombosis. Some physicians completely opposed ovarian stimulation based on their experience of ovarian stimulation failure in women in the puerperium They recommended ovarian tissue cryopreservation after 1 or 2 cycles of chemotherapy, and initial remission. One participant raised the very small risk of the presence of malignant cells in the ovarian tissue in this case.

#### Case 2

A participant posed a clinical question regarding a patient with relapsed Hodgkin’s disease at age 15. She had previously undergone OTC at age 11 prior to commencing chemotherapy which included Adriamycin, Bleomycin sulfate, Vinblastine Sulfate, and Dacarbazine (ABVD). At age 15 she had normal menarche, regular periods and normal follicle-stimulating hormone (FSH) levels. The proposed hemato-oncology treatment plan was autologous bone marrow transplantation. Discussion revolved around the option of an additional fertility preservation procedure. There were 31 responses from 11 participants within 4 h. Members raised different opinions about the estimated low efficacy of OTC in premenarchal girls, and thus suggested ovarian stimulation and oocyte cryopreservation as an additional option. Several clinicians were reluctant to recommend oocyte cryopreservation, given the patient’s young age, clinical situation and limited time for a full cycle, as well as reported high rates of aneuploidy of oocytes of young girls. There was ambivalence regarding counseling the patient and her family given the potential risks and unclear benefit. However the treating doctor counseled the family to consider oocyte cryopreservation.

#### Online survey

**Fig. 1:**
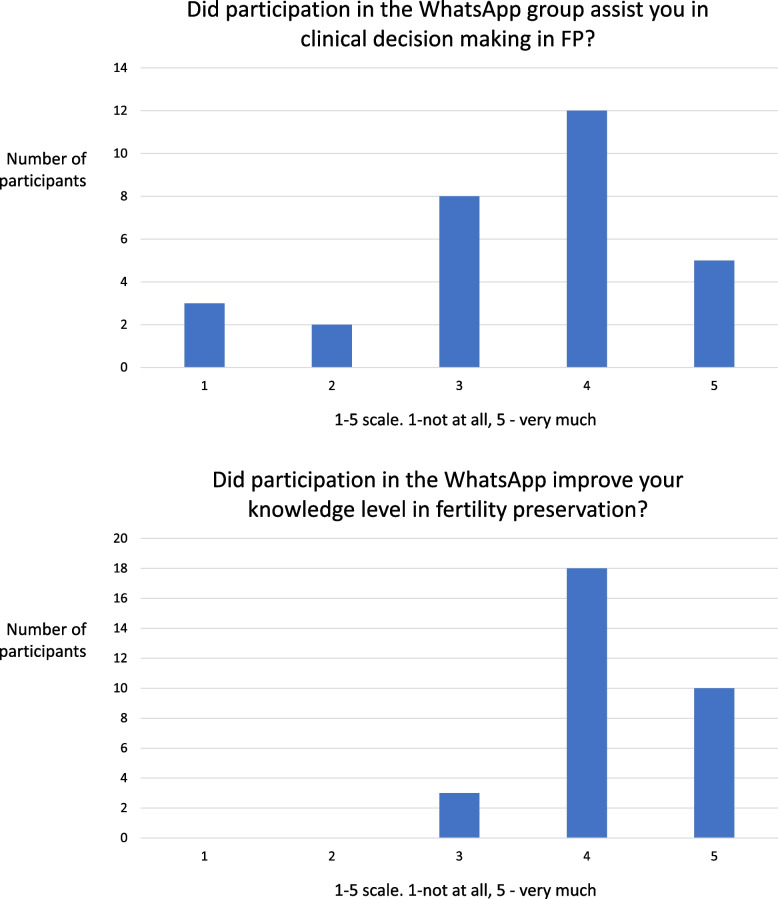
Responses to survey among participants in an online discussion group about fertility preservation (FP). *n* = 31

An anonymous online survey among group members was conducted during August 2022 (*n* = 31, response rate 94%). Participants’ mean clinical experience as board certified obstetrician/gynecologists was 15.7 ± 9 years (range 1.5–40). In response to the question “did participation in the WhatsApp group assist you in clinical decision making in FP”, 58% of respondents rated 4 or 5 on a 1–5 scale, where 1 was ‘not at all’ and 5 was ‘very much’. In response to the question “did participation in the WhatsApp group add to your knowledge in FP”, 90% of respondents rated 4 or 5 on a similar 1–5 scale (Fig. 1). When asked “during the last four years, did you present a query to the group?”, 17 (55%) responded positively. Years of experience were similar for participants who raised questions and for those who did not (14.2 ± 6.8 vs. 17.4 ± 11.2, *p* = 0.44).

A medium correlation and a statistical trend was demonstrated between seniority and 'Assistance in clinical decision making (*r*(29) = 0.342, *p* = 0.06). A small and not statistically significant correlation was found between seniority and 'Knowledge in the field of fertility preservation' (*r*(29) = 0.211, *p* = 0.263).

No statistically significant difference in seniority was found between those who raised questions and those who did not (*M* = 17.4 ± 11.2 vs. *M* = 14.1 ± 6.8, *p* = 0.44).

## Discussion

Data sharing in the modern era involves the use of digital social networks. We evaluated a WhatsApp platform used by Israeli REI physicians for fertility preservation challenges. Content analysis of the dedicated WhatsApp discussion group revealed that this group was rapidly growing and active in aiding physicians to discuss and plan treatment in a wide range of FP cases, for which, published, evidence-based data, is absent. Common issues of concern for FP clinicians included management of oncofertility cases, queries about potential gonadotoxic damage by targeted anti-cancer therapies, and fertility preservation in female patients with premature ovarian insufficiency. We also demonstrated that clinicians found participation in this group useful, including expansion of knowledge and assistance in clinical decision making. This provided an opportunity for a genuine, quantifiable glimpse of real-life concerns of clinicians involved in fertility preservation.

Management of clinical cases of oncofertility were the most frequently discussed issue, prompting a wide range of opinions and clinical options offered by group members. While guidelines offer a general framework for fertility preservation, clinical scenarios often present specific dilemmas requiring prompt solutions. One example is the management of FP immediately after pregnancy, which was discussed in our group during time of analysis. While the first description of a similar case was first published in 2019 [9], actual experience in this situation was shared in the discussion group by clinicians from three centers. This demonstrates how online communication can efficiently summarize clinical approaches and experience of many clinicians when information in standard medical literature is limited.

Interestingly, management of breast cancer, the most common malignancy in women, was raised in only two discussions. We cautiously interpret this finding as reflecting the fact that FP in women with breast cancer has become a mainstream and routinely performed FP service, and therefore raises relatively fewer controversies.

The second most common issue raised was whether FP is indicated before treatment with targeted anti-cancer therapies. Introduction of such agents aims to exclusively affect cancer cells and therefore may have better efficacy and less adverse side effects than classical chemotherapy [4]. However, there is limited data on their potential gonadotoxic effect on humans. Our findings reflect the need for clinicians to make decisions despite uncertainty and lack of knowledge. This reinforces the urgent need for more research in this area [10].

Members also posed queries about management of women and girls with POI. Here, the main issue discussed was the viability of FP given diminished ovarian reserve. This is even more vague and elusive regarding girls, for example with Turner syndrome [11]. Efficacy of OTC in girls with normal ovarian reserve is currently unknown, but will probably be low in girls with ovarian insufficiency, therefore offering OTC in this situation is debatable [12]. On the other hand, women and girls with POI are at high risk to lose their chances for genetic motherhood in the near future, and therefore may prefer OTC even when chances for success are unknown.

The internet has enhanced communication in medicine significantly, for both professional-patient and intra-professional communication. Traditionally, physicians relied on personal communication, local staff meetings or occasional conferences. These are all limited in extent and availability and are time consuming. Asynchronous digital communication offers a platform for timely knowledge sharing and clinical discussion with a community of practice made up of a large number of colleagues who are geographically dispersed. This is especially useful in a relatively new field where the number of specialists practicing in a single center is small. According to our survey, clinicians involved in FP management reported gaining significant knowledge from participating in a dedicated professional WhatsApp group. Moreover, the majority of group members found participation in the group useful for making clinical decisions.

More than half of the participants actively initiated discussions in the WhatsApp group. Members of this group had considerable experience as specialists in obstetrics and gynecology (average 15 years post completion of residency). However, experienced clinicians were not less likely to raise queries, gain knowledge or assistance in clinical decision making than less experienced participants. This highlights the fact that dilemmas in FP are challenging for both highly and less experienced clinicians.

The approach we describe has several limitations. Ideally, medical decisions should be based upon best available research data rather than on expert opinion. Possibly, responses in an online group may be inaccurate, outdated or reflect personal opinion rather than established guidelines of professional groups or peer-reviewed publications. We do not suggest replacing scientific and clinical research with personal viewpoints. However, in situations where good quality evidence is absent, shared knowledge and opinions via intra-professional consultation can assist physicians and their patients.

Further, participants differ in extent of participation in discussion groups. Differences in knowledge, experience and overconfidence or lack of it may all play a role in participation in such professional group. Perhaps some clinicians may feel uncomfortable in raising queries and possibly exposing lack of professional knowledge and experience to their colleagues. Others may be overconfident and present firm opinions which are not necessarily correct. Therefore, opinions and clinical suggestions presented on such a group should be judged cautiously.

This group was initiated as the particular endeavor of colleagues specifically interested in fertility preservation rather than a formal organizational group. Up until the time of analysis of data, there was no intention to reach all relevant institutions in Israel or all REI specialists on a national level. Therefore, we could not provide data on number of clinicians practicing FP or on the number of patients requiring consultation for this issue. However, since in the Israeli health system nearly all oncological treatments as well as medical fertility preservation (i.e., not including planned oocyte cryopreservation to prevent age related fertility loss) are performed in general hospitals, our whatsapp group included clinicians involved in the vast majority of these procedrues in medical FP in the country.

In addition, while most members of the groups found it helpful in clinical decision making, we did not directly compare the whatsapp group to other means of communication such as email or telephone.

In conclusion, we describe a useful internet platform that aids clinicians involved in fertility preservation to deal with common controversial issues. These findings may direct future research and educational endeavors, including creation of guidelines focusing on key, essential issues relevant to FP clinicians. Intra-professional collaboration by whatsapp group adds knowledge and assists in clinical decision making.

### Supplementary Information

**Additional file 1: Supplementary Table 1. **Full list of queries raised in an online discussion group during study period. ABVD - doxorubicin, bleomycin, vinblastine, dacarbazine. AFC – antral follicular count. ALL – acute lymphoblastic leukemia. AMH – anti mullerian hormone. BMT – bone marrow transplantation. BRCA – Breast cancer antigen. FMF – familial Mediterranean fever. FP – fertility preservation. FSH – follicle stimulating hormone. GnRH – Gonadotropin releasing hormone. IBD – inflammatory bowel disease. OTC – ovarian tissue cryopreservation. SLE – Systemic lupus erythematosus.

## Data Availability

The datasets used and/or analyzed during the current study are available from the corresponding author on reasonable request.
