# Supplementary material for: What are the most common controversial clinical issues in fertility preservation? A content analysis of a collaborative professional online consultation group
Source: Reprod Biol Endocrinol. 2023 Aug 24;21:77. doi: 10.1186/s12958-023-01122-5 (PMC10463612; doi:10.1186/s12958-023-01122-5)
Supplement: Supplementary file 1 — Additional file 1: Supplementary Table 1. Full list of queries raised in an online discussion group during study period. ABVD - doxorubicin, bleomycin, vinblastine, dacarbazine. AFC – antral follicular count. ALL – acute lymphoblastic leukemia. AMH – anti mullerian hormone. BMT – bone marrow transplantation. BRCA – Breast cancer antigen. FMF – familial Mediterranean fever. FP – fertility preservation. FSH – follicle stimulating hormone. GnRH – Gonadotropin releasing hormone. IBD – inflammatory bowel disease. OTC – ovarian tissue cryopreservation. SLE – Systemic lupus erythematosus. [file 12958_2023_1122_MOESM1_ESM.docx]

| **Clinical oncofertility questions** |
| --- |
| 15 y.o. 4 years post recovery from Hodgkin’s disease, treated with ABVD. Had OTC for FP. Now – recurrence of HD. Regular menses, day 3 FSH = 7. Should another FP procedure be offered before BMT? |
| Should FP be recommended for a 7 y.o. with Fanconi anemia prior to BMT? |
| Ovarian stimulation with gonadotropins in a woman with endometrial cancer – is co treatment with Letrozole appropriate? |
| 16.5 y.o. with nodular lymphocyte predominant Hodgkin’s disease, planned to receive 2.5 gr of cyclophosphamide. Oncologist opposes FP. what should we recommend? |
| What FP is best in a woman with Hodgkin’s disease one week post delivery? |
| 33.5 y.o. women with stage IV breast cancer, treatment includes Tamoxifen, Herceptin (trastuzumab), Pergeta (pertuzumab)She requests oocyte/embryo cryopreservation. What should we do? |
| What is recommended ovarian stimulation protocol a woman with endometrial cancer receiving high dose progesterone? |
| 25 y.o. underwent ovarian cystectomy due to borderline ovarian tumor 8 years ago. Now has suspected bilateral recurrence. Should we offer FP before or after surgery and histological diagnosis? |
| 17 y.o. – had OTC before chemotherapy for stage IV Ewing sarcoma age 10. Normal puberty and menstruation but ovarian reserve tests (AFC, AMH) are very low. Should we suggest oocyte cryopreservation? |
| Can we use gonadotropins for FP in women with breast cancer prior to scheduled MRI? Does treatment with gonadotropins and elevated hormone levels disturb the interpretation of breast MRI? |
| How early can we start hormonal stimulation for FP after termination of pregnancy? |
| 40 y.o, rectal adenocarcinoma T3N1. Should we recommend transposition of the uterus and the ovaries outside the radiation field? |
| 15 y.o. stage 3 Hodgkin’s disease. How should we perform FP? |
| **Potential gonadotoxicity** |
| Should we offer FP in carcinoma of thyroid prior to radioactive iodine treatment? |
| Is there a risk to future fertility by [Pembrolizumab (Keytruda)?](https://www.keytruda.com/) |
| Is there a risk to future fertility by Ibrance (Palbociclib)? |
| Patient with IBD and FMF presented with ovarian failure following treatment with Azathioprine (Imuran). Has anyone had a similar observation? |
| 17 y.o. Recurrent thyroid cancer treated twice already with radioactive iodine. Now she has recurrence and is planned to receive another treatment. Should we offer FP? |
| Is Cladribine gonadotoxic? |
| 23 y.o., stage IV clear cell carcinoma of kidney. Planned to receive Pembroizumab and Lenvatinib. Should we offer FP? |
| Is adalimumab (Humira) for Rheumatoid arthritis gonadotoxic? |
| Is Capecitabine (Xeloda) gonadotoxic? |
| **Premature Ovarian Insufficiency (POI)** |
| 15 y.o. with POI: FSH 132, AMH 0.4. Should we offer FP? |
| POI due to Azathioprine (Imuran) in patient with IBD and FMF?. Should we offer FP? |
| 19 y.o. Irregular menses, elevated FSH (23,17,16) with low E2 in several tests, but AMH 7.4 and AFC is high. Should we offer FP? |
| What AMH normograms are most reliable? |
| 12.5 y.o., Turner Syndrome mosaicism, low ovarian reserve markers. What should we recommend? |
| When should we recommend FP in Turner mosaicism? |
| **Ovarian Tissue Cryopreservation (OTC)** |
| Is it safe to transplant ovarian tissue to a girl with ovarian failure that was removed 7 years ago during remission from ALL prior to BMT? staining of ovarian tissue are negative for ALL markers |
| Should we freeze ovarian cortex when removing streak ovaries in ovarian dysgenesis? |
| How long can we keep ovarian tissue in media prior to freezing? |
| **Non-oncological FP issues** |
| 38 y.o, with bilateral large ovarian cysts (10 & 7 cm). Should we offer FP prior to surgery? |
| 19 y.o. had unilateral oophorectomy due to ovarian torsion. Should we offer FP? |
| Is FP recommended prior to cyclophosphamide for a patient with SLE? |
| **other issues** |
| in cases of medically indicated FP - should we offer oocyte cryopreservation as well as embryos? |
| Is GnRH agonist for ovarian protection recommended in cases other than breast cancer? |
| Is Cannabis safe or does it affect outcome of oocyte cryopreservation cycle? |
| 28 y.o., single male. Stage IV Hodgkin’s disease with superior vena cava syndrome. Very few immotile spermatozoa in ejaculated sperm Is testicular sperm retrieval recommended prior to highly gonadotoxic treatments? |

Supplementary table 1: full list of queries raised in an online discussion group during study period. ABVD - doxorubicin, bleomycin, vinblastine, dacarbazine. AFC – antral follicular count. ALL – acute lymphoblastic leukemia. AMH – anti mullerian hormone. BMT – bone marrow transplantation. BRCA – breast cancer antigen. FMF – familial Mediterranean fever. FP – fertility preservation. FSH – follicle stimulating hormone. GnRH – gonadotropin releasing hormone. IBD – inflammatory bowel disease. OTC – ovarian tissue cryopreservation. SLE – systemic lupus erythematosus
